# The Developing Story of Predictive Biomarkers in Colorectal Cancer

**DOI:** 10.3390/jpm9010012

**Published:** 2019-02-07

**Authors:** Stergios Boussios, Mehmet Akif Ozturk, Michele Moschetta, Afroditi Karathanasi, Nikolaos Zakynthinakis-Kyriakou, Konstantinos H. Katsanos, Dimitrios K. Christodoulou, Nicholas Pavlidis

**Affiliations:** 1 Acute Oncology Assessment Unit, Medway NHS Foundation Trust, Windmill Road, Gillingham ME7 5NY, Kent, UK; a.karathanasi@nhs.net; 2 AELIA Organization, 9th Km Thessaloniki—Thermi, 57001 Thessaloniki, Greece; 3 Department of Internal Medicine, Bahcesehir University School of Medicine, 34353 Istanbul, Turkey; ozturkakif@yahoo.com; 4 Drug Development Unit, Sarah Cannon Research Institute, 93 Harley Street, London W1G 6AD, UK; michelemoschetta1@gmail.com; 5 Leicester Diabetes Research Centre, Gwendolen Road, Leicester LE5 4PW, UK; nikolaoszakynthinakis@gmail.com; 6 Department of Gastroenterology, University Hospital of Ioannina, Faculty of Medicine, School of Health Sciences, University of Ioannina, 45110 Ioannina, Greece; khkostas@hotmail.com (K.H.K.); dchristodoulou@gmail.com (D.K.C.); 7 Medical School, University of Ioannina, Stavros Niarchou Avenue, 45110 Ioannina, Greece; npavlid@uoi.gr

**Keywords:** colorectal cancer, biomarkers, prognostic and predictive markers, treatment resistance

## Abstract

Colorectal cancer (CRC) is the third most common malignancy worldwide. Surgery remains the most important treatment for non-metastatic CRC, and the administration of adjuvant chemotherapy depends mainly on the disease stage, which is still the strongest prognostic factor. A refined understanding of the genomics of CRC has recently been achieved thanks to the widespread use of next generation sequencing with potential future therapeutic implications. Microsatellite instability (MSI) has been suggested as a predictive marker for response to anti-programmed-cell-death protein 1 (PD-1) therapy in solid tumors, including CRC. It should be noted that not all cancers with MSI phenotype respond to anti-PD-1 immunotherapy, highlighting the urgent need for even better predictive biomarkers. Mitogen-Activated Protein Kinase (*MAPK*) pathway genes *KRAS*, *NRAS*, and *BRAF* represent important molecular targets and could serve as independent prognostic biomarkers in CRC, and identify those who potentially benefit from anti-epidermal growth factor receptor (EGFR) treatment. Emerging evidence has attributed a significant role to inflammatory markers including blood cell ratios in the prognosis and survival of CRC patients; these biomarkers can be easily assessed in routine blood exams and be used to identify high-risk patients or those more likely to benefit from chemotherapy, targeted therapies and potentially immunotherapy. Analysis of cell-free DNA (cfDNA), circulating tumor cells (CTC) and/or micro RNAs (miRNAs) could provide useful information for the early diagnosis of CRC, the identification of minimal residual disease and, the evaluation of the risk of recurrence in early CRC patients. Even the selection of patients suitable for the new targeted therapy is becoming possible with the use of predictive miRNA biomarkers. Finally, the development of treatment resistance with the emergence of chemo-resistance clones after treatment remains the most important challenge in the clinical practice. In this context it is crucial to identify potential biomarkers and therapeutic targets which could lead to development of new and more effective treatments.

## 1. Introduction

Colorectal cancer (CRC) is the third most commonly diagnosed cancer and the fourth leading cause of cancer mortality worldwide [1]. The estimated incidence for 2018 is over 140,000 new cases and with mortality of over 50,000 annually in the United States [2]. Survival has increased in the last 30 years after the introduction of screening programs and the development of new target agents; however, the 5-year relative survival from CRC remains only 68%, calling for development of new and more effective treatments [3]. Of note, among patients who undergo curative surgery for CRC, approximately one third will experience disease recurrence underscoring the importance of developing biomarkers to identify these patients potentially suitable for post-surgery treatment intensification [4]. The estimated 5-year survival in the metastatic setting is only around 13% [5]. Risk factors associated with the incidence of CRC include older age, male sex, lifestyle, inflammatory bowel disease and a previous personal history of CRC. It is mostly sporadic, though approximately 20–30% of patients carry inherited mutations [6,7]. Accumulation of numerous genetic mutations and/or epigenetic changes is required to drive the carcinogenic progression. CRC development follows a multistep path; it begins as benign adenomatous polyps on the inner wall of the colon and rectum in the large intestine and progressively develops into advanced adenoma and carcinoma in situ, invasive carcinoma and eventually distant metastases that represent the most advanced stage of development [8]. 

Treatment of CRC usually involves surgical resection of the primary tumor followed by chemotherapy and/or targeted therapy for the advanced stage disease [9]. Despite these advancements, as mentioned above, drug resistance still remains a widely unresolved issue [10]. Several mechanisms drive drug resistance development in CRC, including limitation of drug transport within the tumor cells, dysregulation of cellular physiology, and reduction of drug sensitivity through epigenetic modifications or dysregulation of miRNA levels [11]. Additional mechanisms include apoptosis, drug metabolism, DNA damage repair dysfunctions and dysregulation of cell cycle checkpoints [12]. With this regard, there is an urgent need for personalized treatment based on molecular biomarkers, in conjunction with the use of baseline clinical and laboratory variables. 

Specific genomic mutations serving as predictive biomarkers are examined in formalin fixed tumor tissues. Blood-based biomarkers are appealing given that blood is a readily available, inexpensive, and minimally invasively obtained, which allows for repeated sampling. Among several major genetic mutations in CRC, the RAS mutational status (*KRAS* or *NRAS* mutations in exons 2, 3, or 4) has been recognized as an important biomarker able to predict response to anti-EGFR antibodies [13]. It has also been reported that RAS mutation is correlated with the oncological aggressiveness of CRC [14], the site-specific risk of recurrence [15], and the pathologic response to chemotherapy [16]. There is growing evidence that inflammation drives development of this disease [17]. As a result, many studies have investigated the predictive and prognostic role of various blood based inflammatory markers, including neutrophil–lymphocyte ratio (NLR), lymphocyte–monocyte ratio (LMR), and platelet–lymphocyte ratio (PLR) [18,19,20]. miRNAs have crucial regulatory functions, including regulation of important cellular functions like proliferation, apoptosis, angiogenesis, and immune response [21]. They have been shown to have roles as tumor suppressor genes and oncogenes, and their diagnostic, prognostic, and predictive implications are now being explored. Both plasma and serum are suitable for investigations of miRNAs as blood-based biomarkers [22]. 

This review focuses on KRAS, NRAS, BRAF, human epidermal growth factor receptor 2 (HER2) amplification, and miRNAs as prognostic and predictive biomarkers. Risk stratification by primary tumor site and assessment of tumor laterality in patient selection for EGFR antibody treatment are also considered. 

## 2. DNA Mismatch Repair Genes and Microsatellite Instability

Microsatellite instability is caused by mutations in the mismatch repair gene (*MMR*) with the consequent inability to correct DNA errors that take place during cell replication. Mismatch repair genes are inactivated either as a result of sporadic *MLH1* promoter hypermethylation, or germline mutations in *MLH1*, *MSH2*, *MSH6* and *PMS2* genes [23]. It is now recommended that *MSI* status should be evaluated in all newly diagnosed CRC cases. This important clinical information with prognostic value for stage II CRC can be used as a screening marker to identify Lynch syndrome patients, and may predict response to immunotherapy in patients with stage IV disease [24]. 

In a study, it has been demonstrated that immune cell PD-L1 expression was significantly higher in MSI-H CRC as compared to MMR-proficient (MSI-L) tumors, with no differences among the different MSI-H molecular subtypes [25]. In a phase II study, patients with MSI-H colon cancer treated with the anti PD-L1 antibody pembrolizumab reported an objective response rate (RR) of 62% as compared to MSI–L tumors where the RR was 0% [26]. The high lymphocyte infiltration and the increased expression of neoantigens in MSI-H CRC, and other tumors, consequently to their high genomic instability can explain this observation [27]. Of note, pembrolizumab is now approved in patients with advanced cancer and MSI-H status in a tissue agnostic fashion. It has been showed that neoantigens induce an active immune microenvironment featuring two opposing forces; an immune stimulatory force represented by increased cytotoxic effector T lymphocytes and an immune inhibitory force including upregulated PD-1/PD-L1 checkpoints. Some MSI-L tumors harbored paradoxically high tumor-infiltrating lymphocytes, resulting in high immune cell PD-L1 expression as well; however, this correlation is not as direct as in the case of MMR-deficient (MSI-H) tumors [25]. Another novel monoclonal antibody that targets PD-1 checkpoints and boosts the immune response against cancer cells is nivolumab. The findings of the CheckMate 142 study suggest that nivolumab is a promising therapeutic option for patients with previously treated MSI-H metastatic CRC [28]. In this study, MSI-H CRC patients received nivolumab or 3 mg/kg nivolumab plus 1 mg/kg ipilimumab for four doses followed by 3 mg/kg nivolumab every two weeks until disease progression, or unacceptable toxicity. The RR for MSI-H patients receiving 3 mg/kg nivolumab was 25.5%, as compared to 33.3% for the combination arm. The six-month disease free survival (DFS) rate was 45.9% (95% Confidence Interval [CI]: 29.8–60.7) and 66.6% (95% CI: 45.5–81.1) for nivolumab and nivolumab plus ipilimumab, respectively. The six-month overall survival (OS) rate was 75% (95% CI: 58.5–85.7) and 85.1% (95% CI: 65.0–94.2), respectively. Furthermore, the combination therapy of nivolumab plus ipilimumab was well tolerated and, as such, could be a reasonable therapeutic option in MSI-H CRC. Ongoing studies are evaluating the combination of nivolumab with other immunotherapies in MSI-H metastatic CRC.

The use of adjuvant chemotherapy in patients with stage II CRC remains controversial, in terms of the potential benefits versus risks of treatment. Current evidence supports the use of MMR testing for the implication of adjuvant therapy in this subset of patients. The favorable prognosis of patients with stage II MSI-H CRC and the lack of benefit from adjuvant 5-FU-based therapy, indicate that these patients should avoid adjuvant chemotherapy. Therefore, testing for MMR status by MSI analysis or immunohistochemistry should be recommended in stage II CRC in patients where adjuvant treatment is a consideration. On the other hand, the investigation of potential benefits of the current standard FOLFOX regimen (5-fluorouracil, leucovorin and oxaliplatin) in stage III MSI-H CRC subgroup has not been completed. Currently, available data do not justify the exclusion of patients with stage III MSI-H CRC from adjuvant treatment with FOLFOX, since the responsiveness of these tumors to oxaliplatin or FOLFOX has not been confirmed. As such, MMR status in patients with stage III disease is of research interest. However, the use of MMR status represents a further step toward personalized cancer care. 

## 3. BRAF Mutations 

In the *RAS* signaling pathway, *BRAF* is the direct downstream target of *KRAS* [29]. *BRAF V600E* mutation is present in approximately 10% of CRC patients, which is mutually exclusive of the *KRAS* mutations found in 35–45% of cases [30]. The majority of these consist of a substitution of glutamic acid for valine at the V600 hotspot in exon [31]. *BRAF* mutations are more frequent in female sex, older age, right colon and proximal tumors with poor differentiation, mucinous histology and infiltrating lymphocytes, which are usually MSI-H [32]. The presence of *BRAF* mutation is associated with a shorter DFS and OS in MSI-L tumors. Interestingly, this mutation results in an improved and longer DFS in MSI-H tumors; however, OS has been shown to be not significantly affected. *BRAF* mutation can also represent a therapeutic target in metastatic CRC but targeting *BRAF* will also require the blockade of other pathways including, *EGFR*, *MEK* and *PI3K* differently from melanoma and non-small cell lung cancer where *BRAF* inhibition produces therapeutic benefits [33]. Table 1 lists the activity of *BRAF* inhibitors and combinations of targeted therapies in *BRAF V600E*-mutated CRC [34,35,36,37,38,39,40,41,42,43]. However, it has also been reported that *EGFR* inhibitors provide limited benefit in patients with *BRAF V600E* metastatic CRC [44]. SWOG 1406 assessed irinotecan-cetuximab combination as a control arm in the second or third line setting in *BRAF V600E* metastatic CRC, and demonstrated an RR of 4% and median DFS of two months, which confirm the limited activity of cetuximab in this population [45]. It remains controversial whether patients harboring *BRAF V600E* mutation should not receive an anti-EGFR treatment in the first line setting. In this context, a molecular subgroup analysis of the TRIBE trial suggests that fit patients can achieve better OS with the combination of 5-fluorouracil, oxaliplatin, and irinotecan (FOLFOXIRI)-bevacizumab compared to irinotecan, 5-fluorouracil (FOLFIRI)-bevacizumab [46] but prospective studies are needed to address this issue.

Despite the fact that BRAF inhibitors have a high response rate in *BRAF V600* mutant melanoma, and non-small cell lung cancer, their efficacy as monotherapy in *BRAF V600E* CRC is limited [36]. It has been supported that high levels of basal receptor tyrosine kinase signaling in CRC underlie rapid adaptive resistance taken that extracellular signal-regulated kinase (*ERK*) inhibition releases *EGFR* from negative feedback suppression [47]. Receptor reactivation leads to rebound in *ERK* phosphorylation by either reactivating *ERK* signaling, or recruiting *RAS* which forms *RAF* dimers, which are resistant to *RAF* inhibitors. With this regard, they have been investigated combined approaches of *BRAF* and *EGFR* inhibitors with response rates of 10–25% [39,48]. However, the addition of an *MEK* inhibitor to *RAF* and *EGFR* inhibitors contributes to better inhibition of *ERK* signaling. As such, response rates are further improved [34]. Phase 3 BEACON trial currently evaluates whether doublet or triplet targeted therapy affects better survival as compared to chemotherapy with irinotecan and cetuximab in the cohort of *BRAF V600E* metastatic CRC. Preliminary results for the triplet combination of encorafenib (*RAF* inhibitor), binimetinib (*MEK* inhibitor), and cetuximab demonstrated overall RR of 48% in this subset of patients [49].

Despite the fact that overall prognosis for *BRAFV600E* mutant metastatic CRC is worse as compared to *BRAF*-wild-type CRC, the assessment of the *BRAF V600E* status should be mandatory in future adjuvant trials with the prospect to be incorporated in the clinical practice. Even though there is evidence of response to combined *BRAF/EGFR* and *BRAF/MEK* inhibition, the reported overall RR and median DFS should be further improved and there is still need for optimization of this therapeutic strategy. Access to combinations of *BRAF*, *MEK*, and other pathway inhibitors is currently not Food and Drug Administration (FDA) approved for the treatment of metastatic *BRAF V600E* CRC; nevertheless, this off-label strategic implementation deserves consideration based on the growing body of literature. Overall, taken the poor prognosis and distinct clinical features of metastatic *BRAFV600E* CRC, earlier identification of the mutation may expand therapeutic, including clinical trials, options prior to patients’ deterioration.

## 4. Mutations in the RAS Gene 

*KRAS* proto-oncogene encodes the GTPase protein *KRAS*, which has a pivotal role in several molecular pathways, lies downstream of EGFR and potentially engages effectors that control proliferation, differentiation, and survival [50]. Fifteen percent of CRC carry mutations in exons 2, 3 and 4 of the *KRAS* gene [51]. *KRAS* mutations are associated with a shorter DFS and OS in patients with MSI-L tumors, which represent approximately 90% of all stage III colon cancer but have no prognostic implication in patients with MSI-H tumors [52]. Additionally, MSI-L tumors are located in the distal colon while the majority of cases with MSI-H tumors develop in the proximal colon [53]. It has been demonstrated that, among CRC patients with point mutations in exon 2 (codons 12 and 13) or exon 3 of *KRAS*, prognosis is worse only in those having a distal tumor [54]. As previously mentioned, KRAS hotspot mutations in exons 3 and 4 and in *NRAS* are predictive biomarkers for EGFR therapy benefits [13], and the use of EGFR inhibitors in patients with RAS mutant tumors may even lead to a shorter survival [13].

The prognostic role of *KRAS* mutation was not clarified until recently. Two trials supported the prognostic importance of *KRAS* mutation for the subset of patients with CRC. The late 1990s RASCAL study [55] demonstrated that *KRAS* mutation increases the risk of recurrence and death, predominantly in a guanine to thymine mutation. Furthermore, in the expanded RASCAL II study, the prognostic role of the *KRAS* mutation, just limited to a glycine to valine mutation, was detected in 8.6% of the participants, and affected statistically significant both DFS (*p* = 0.004, hazard ratio [HR] = 1.3) and OS (*p* = 0.008, HR = 1.29) [56]. On the other hand, phase III translational study of PETACC3 [32], revealed that the *KRAS* mutation status does not have an important prognostic impact on stage II and III CRC. The difference in results may potentially be explained by the difference in sample size.

Two large, randomized phase III clinical trials established the predictive value of *KRAS* for anti-EGFR treatment [57,58]. Panitumumab and cetuximab were compared to best supportive care in patients with CRC refractory to chemotherapy. The response rate of panitumumab was 17% and 0% for the wild-type *KRAS* and the mutant cohort, respectively (*p* < 0.0001) [58]. Moreover, the combined chemotherapy—either FOLFIRI or FOLFOX—with the anti-EGFR antibodies cetuximab or panitumumab was related to the achievement of better RR, DFS or OS alone in the wild-type KRAS group, independently of the treatment line [59,60,61]. In the CRYSTAL study [59], patients were randomized to receive cetuximab plus FOLFIRI, versus FOLFIRI alone. The HR for DFS in the cetuximab–FOLFIRI group as compared with the FOLFIRI group was 0.85 (95% CI, 0.72 to 0.99; *p* = 0.048). There was no significant difference in the OS between the two arms (HR, 0.93; 95% CI, 0.81 to 1.07; *p* = 0.31). In contrast, there was a significant interaction between treatment group and *KRAS* mutation status as regarding the tumor response (*p* = 0.03). The benefit of cetuximab appeared to be restricted to patients without mutations in the KRAS gene. The hazard ratio for DFS among patients with wild-type *KRAS* tumors was 0.68 (95% CI, 0.50 to 0.94), in favor of the cetuximab–FOLFIRI group. OPUS study [62], evaluated whether the RR of cetuximab combined with FOLFOX was superior to FOLFOX alone in the first-line treatment of metastatic CRC. The impact of *KRAS* mutation status was assessed. In the wild type *KRAS* cohort, the addition of cetuximab to FOLFOX led to a 2.54-fold increased response as compared to FOLFOX alone (61% vs. 37%). On the other hand, for patients with *KRAS* mutations, the RR for cetuximab plus FOLFOX was lower than the FOLFOX alone (33% vs. 49%). Finally, a meta-analysis of 11 studies [63] revealed a significant treatment effect interaction between KRAS status and addition of anti-EGFR antibodies to standard chemotherapy for both DFS (95% CI: 57–90%, *p* = 0.005) and, RR difference (95% CI: 8.22%, *p* < 0.001).

There are still no available drugs currently targeting the activating mutations of *KRAS*. This represents a major therapeutic problem, as *KRAS* mutations are associated with dismal prognosis among CRC patients. It seems that there will be a long and winding way until the first approved KRAS inhibitor, mainly due to the genetic heterogeneity of the *KRAS*-mutant disease. Novel and effective approaches are currently under investigation, for targeting KRAS. Identification of molecules that could either bind the mutated sites of *KRAS*, or inhibit the synthesis at the DNA level of the mutated protein, is in progress. There is an effort for developing small molecules binding to *KRAS G12D* mutant, and preventing the formation of active KRAS-GTP [64]. miRNAs could be a further therapeutic potential to explore effective targeting of KRAS-mutant CRC. miRNAs regulate critical pathways involved in the CRC pathogenesis, including the p53, PI3K, RAS, MAPK, EMT transcription factors, and Wnt/β-catenin pathways [65]. As such, the development of effective drugs for targeting complicated pathways in CRC remains challenging.

## 5. The Epidermal Growth Factor Receptor Family 

The crucial role of EGFR signaling in the survival of several tumors, including CRC, is supported by the approval of anti-EGFR targeted therapies; unfortunately, resistance to target therapy inevitably emerges; HER2 has recently been shown to represent one potential resistance mechanism leading to anti-EGFR antibody therapy resistance in *KRAS* wild type tumors [66]. 

*HER2* amplification has been detected in approximately 5% of KRAS wild type cancers. Therapeutically, combination treatment with trastuzumab and lapatinib has resulted in a 35% overall RR, and a median DFS of approximately 5.5 months in heavily pretreated patients harboring HER2-amplified CRC. These findings support the role of HER2 expression as a predictive biomarker of anti-HER2 treatment response in this subset of metastatic CRC [41]. Furthermore, in a cohort of KRAS wild type metastatic CRC patients treated with anti-EGFR treatment, those with higher expression and activation of *EGFR* and *HER3* membrane receptors had a better OS, independently of the line of treatment [67]. EGFR pathway activation is associated with a better OS in KRAS wild type metastatic CRC patients receiving anti-EGFR treatment. This could be explained by the fact that these tumors are more dependent on EGFR signaling and thus more sensitive to its inhibition. 

In *KRAS* wild type patients, the PI3K pathway can explain the variability of response to anti-EGFR therapies; in this subset of tumors, several potential mechanisms could be at the origin of the EGFR activation, including the overexpression of *EGFR* ligands and specifically epiregulin (EREG) and amphiregulin (AREG). This has been associated with a better response to anti-EGFR therapy in *KRAS* wild type CRC [68]. FIRE1 clinical trial concluded that high EREG mRNA levels could represent a positive prognostic marker for both DFS and OS, whereas high AREG levels did not affect the outcome in a statistical significant way [69].

Apart from KRAS status, and resistance to anti-EGFR therapy, mutations in PIK3CA are associated with proximal colonic tumors, and adverse outcomes for patients with *BRAF* wild-type tumors [70,71]. In patients with stage III colon cancer, PIK3CA mutations in the exon 20 have been shown to have a worse outcome [72], while patients harboring both exon 9 and 20 mutations exhibit a worse prognosis compared to those with *PIK3CA* wild-type or with *PIK3CA* mutation in either of the exons [73]. Interestingly enough, some reports supported the use of aspirin from CRC patients with *PIK3CA* mutations, based on the 29% mortality reduction compared to wild type PIK3CA tumors. As such, PIK3CA mutation in CRC has been reported as a predictive molecular biomarker for adjuvant aspirin therapy [74]; however, confirmatory prospective studies need to be carried out to confirm this hypothesis.

The lack of association between *EGFR* protein expression by immunohistochemistry and response to EGFR-targeted agents is likely due to several technical reasons. Immunohistochemistry is not a strictly quantitative method, which has a substantial impact on the determination of EGFR immunoreactivity. Furthermore, *EGFR* expression might differ between primary tumors and metastatic sites. As such, the evaluation of *EGFR* expression in the primary tumor may not be suitable for predicting the treatment response of metastases. In addition, there is no correlation between EGFR protein expression and *EGFR* gene amplification. Based on this evidence, cetuximab and panitumumab have been approved by the FDA without the need for *EGFR* testing, as a second and third line therapy for advanced CRC. 

Several clinical trials with a second or first generation of tyrosine kinase inhibitors are ongoing. In metastatic settings, tyrosine kinase inhibitors could have a role as maintenance treatment. The combination of different targeted therapies in order to overcome tumor resistance is reasonable. There is evidence that involves different molecular networks as far as resistance to targeted therapies against one pathway is concerned. Several approaches targeting the *EGFR* and its downstream pathways exist. There is urgent need for further biomarkers in both clinical practice and the process of drug development to make prediction of responses to different targeted therapies feasible.

## 6. P53, APC and β-Catenin in CRC Progression

TP53 protein, encoded by a *TP53* tumor suppressor gene, has an important cell proliferation regulatory role and mediates cell growth arrest, DNA repair and apoptosis, following oxidative stress, and cell aberrant proliferation. *TP53* mutation is observed in 50–75% of CRC [8]. MiR-34a is one of the most relevant downstream effectors of p53, and may represent an important biomarker and therapeutic target in CRC, considering that its expression is significantly decreased in CRC patients [75]. It has been shown that *p53*-dependent expression of miR-34a blocks the IL-6R/STAT3/miR-34 feedback loop, resulting in inhibition of tumor progression in CRC [76]. 

Germline adenomatous polyposis coli (APC) mutations represents the genetic basis of familial adenomatous polyposis (FAP); more than 60% of sporadic CRC cases have genetic alterations in the *APC* gene leading to stimulation of the Wnt/β-catenin pathway, which drives tumor initiation and recurrence [8]. APC promoter hypermethylation has also been recognized as an additional cause leading to APC silencing [77]. 

MiRNAs have also been shown to regulate the Wnt pathway in CRC, while activation of the Wnt pathway leads to the increase of expression of miRNAs that directly bind to their gene promoters. MiR-224 has been recently shown to activate the Wnt/β-catenin signaling and direct the nuclear translocation of beta-catenin in CRC [78]. Knockdown of miR-224 inhibits Wnt/β-catenin mediated cell metastasis and cell proliferation. Furthermore, overexpression of miR-101 in CRC reduces beta-catenin nuclear localization and consequently inhibits cancer stem cell-related gene expression [79]. Thus, pharmacological restoration of miR-101 may be considered as a novel therapeutic approach for the prevention of recurrence of CRC. In addition, miR-135a/b targets the 3’UTR of APC, suppresses the expression of *APC* gene, and finally activates the downstream Wnt pathway to promote tumor progression. Overexpression of miR-135b is associated with advanced disease [80].

The predominance of p53 mutations in the major subset of CRC, which is related to the APC inactivation, suggests that the ability of wild type *p53* to restrain the deregulated wild type β-catenin is an essential component of its tumor suppressor function. This effect contributes to the antiproliferative effects of *p53* and probably facilitates *p53*-mediated apoptosis. *P63* and *p73* are functionally and structurally related to p53, and may participate in tumor suppression. However, loss of *p63* and *p73* has not been detected in CRC pathogenesis. Overall, APC and p53 mutations do not seem to be clinically significant. 

## 7. DNA Polymerase Epsilon

Germline mutations in DNA polymerase 1 (POLE) and d (POLD1) contribute to an increased risk of either multiple colorectal adenomas, or CRC. Typically, carriers of POLE mutations are mostly men, and younger, presenting with right-sided colorectal tumors. They only represent around 1% of all CRC, and, as such, the identification of an accurate clinical phenotype has not yet been achieved. There is an urgent need for the establishment of specific criteria for POLE and POLD1 exonuclease mutation screening, in order to optimize the therapeutic approach of patients with these kinds of mutations. 

POLE-mutated CRC is characterized by elevated CD8+ lymphocyte infiltration and the presence of cytotoxic T-cell markers, similarly to immunogenic MSI-H cancers [81]. POLE mutations designate a subset of CRC with more favorable outcomes, based on tumor immunogenicity. The rationale of an immune checkpoint blockade would be therapeutically challenging for carriers of POLE mutations in metastatic settings, and requires further investigation [82].

The definitive implications of POLE proofreading domain mutations in CRC await definition. The evidence of the prognostic effect of POLE proofreading domain mutations in CRC eight years following their discovery is impressive [83], taken the required three decades for the validation of MMR deficiency as a prognostic biomarker [84]. Evaluation of POLE mutation promises to refine risk stratification in CRC. This may lead to identification of a subgroup of patients who will experience benefits by immune checkpoint inhibitors. The rationale of the future studies should be to confirm the favourable prognosis of POLE-mutant CRC independently of the postoperative chemotherapy. As the frequency of POLE mutation in CRC is modest, the validation of this novel biomarker is a challenging requirement.

## 8. Blood Biomarkers 

Circulating tumor cells (CTCs) are identified in the peripheral blood based either on the level of epithelial surface marker expression or on physical features of cancer cells [85]. Their presence indicates active disease, proliferation and metastatic potency, and is followed by genomic analyses that provide data in terms of the tumor biology and real-time monitoring of the therapeutic efficacy [86]. The prognostic value of CTC in early stages of the disease has been established by several studies [87]. However, comparison of their findings seems difficult due to the variety of patients’ population, analytical techniques and timing of specimen collection. 

Indeed, both timing and the site of sampling are factors that influence the outcome of CTC analysis. Regarding this, a meta-analysis of 14 studies revealed that there is a role of CTCs as predictors of recurrence in six out of nine studies in which blood samples were collected at least 24 h postoperatively [88]. In contrast, the prognosis was not associated with perioperative CTC levels. However, an analysis of 12 studies with the participation of patients who underwent colectomy for diseases staged I–III demonstrated that CTC presence was strongly correlated with both poor OS (HR = 3.07, 95% CI 2.05–4.624), and decreased DFS (HR = 2.58, 95% CI: 2.00–3.32), independently of the timing of specimen collection [89]. Furthermore, in a review of 36 studies, the detection of CTC was correlated with poor prognosis in CRC patients, when they were collected in peripheral blood rather than in mesenteric portal blood or bone marrow (HR DFS 3.06, 95% CI 1.74–5.38; HR OS: 2.70, 95% CI 1.74–4.20) [90]. 

Preoperative CTC identification represented the most powerful prognostic factor in early stage disease (HR, 5.5; 95% CI, 2.3–13.6), according to the results of a study enrolled stage I–III CRC patients [91]. This was not the case for postoperative levels of CTC, which are typically much lower, and not associated with survival, neither DFS nor OS. A large study that enrolled stage III CRC patients detected the presence of minimum one CTC only in 35% of the patients [92]. 

The cfDNA releases by cancer cells, and refers to degraded DNA fragments released to the blood plasma or other biological fluids [93]. Analysis of cfDNA, for mutations and genetic aberrations provides real-time monitoring of tumor progression, particularly in stage II resected colon cancer [94,95]. Currently, improved PCR-based methods were implemented to identify genomic alterations in cfDNA [96]. An Alu-based quantitative-PCR could differentiate tumors from adenomas, based on evaluation of cfDNA levels [97]. Aberrant patterns of methylation have been described in the cfDNA [98]. The methylation status of the Septin 9 gene promoter has been used for the diagnosis of early CRC, and the reported sensitivity and specificity is 30–75%, and 90%, respectively [99].

Different studies demonstrated that higher levels of total cfDNA or mutant ctDNA correlate with a shorter OS in patients with metastatic CRC [100,101,102]. The RAS tumor mutational status can be evaluated through analysis of the cfDNA, alternatively to tissue testing. In addition, cfDNA analysis provides information on the mechanisms of acquired resistance to anti-EGFR drugs in metastatic CRC patients [103]. This is likely to be multi-clonal rather than monoclonal. Taking the heterogeneity of metastatic CRC, cfDNA analysis is a strategy that can depict the complex landscape of genetic alterations that lead to resistance to targeted agents. There is still not established evidence for the implementation of cfDNA analysis with regard to treatment decision in clinical practice. Due to a lack of prospective clinical trials, the potential detection of somatic mutations in cfDNA should not modify the treatment.

Liquid biopsy may be a novel approach toward identification of the minimal residual disease in patients with resected CRC. However, the obtained prognostic information through detection of clonal mutations should be translated into sophisticated therapeutic options for the improvement of survival. At the present time, plasma testing has a limited use for *RAS* mutation in CRC. The possibility of false negative result of a *RAS* test on plasma is not rare. However, it may provide complementary information to histology, particularly in patients with recurrence disease following resection of the primary tumor [104].

The use of blood biomarkers in CRC is still extremely limited in clinical practice. Several reasons, including low level of standardization of the tests used, limited number of patients enrolled, and the lack of clear clinical benefit, explain this statement. The importance of liquid biopsy to the current screening policy will be upgraded with the design of prospective studies.

## 9. Blood Cell Ratios

The predictive value of various blood cell ratios in CRC has been supported by several studies. NLR indicates the balance between the inflammatory response and the antitumor immunity. Elevated pre-treatment NLR has been shown to be strongly associated with poor OS (HR 2.17, 95% CI 1.82–2.58) and DFS (HR 1.96, 95% CI 1.64–2.35) in a cohort of CRC patients with liver metastases [105]. The negative predictive value of high NLR has been demonstrated both in locally advanced and metastatic tumors [18]; however, predictive impact of high NLR was not specified in patients diagnosed with stage I and II disease [106]. The prognostic importance of LMR was evaluated in several settings. In a study of chemo-naïve metastatic CRC patients, those with elevated LMR (≥3.11) prior to chemotherapy achieved better DFS (9.2 vs. 7.6 months, *p* < 0.001) and OS (19.4 vs. 16.6 months, *p* < 0.001) as compared to cohorts with low LMR, respectively. These results were reproducible in operable cases of CRC [107]. In addition, a large study demonstrated that low LMR is related to more advanced, poorly differentiated disease, and right-sidedness, whereas high LMR was related to early stages, left-sided tumors and improved OS [19]. In addition, the predictive role of LMR was evaluated in metastatic setting with liver’s involvement, treated with radiofrequency ablation [108]. Patients with LMR > 3.96 reached median OS of 55 months, whereas those with LMR ≤ 3.96 achieved OS of only 34 months, which is statistically significant (*p* = 0.007). Finally, a meta-analysis, included 15 studies, concluded that increased PLR was strongly correlated with shorter OS (pooled HR, 1.53; 95% CI, 1.24–1.89; *p* ≤ 0.001), DFS (pooled HR, 1.68; 95% CI, 1.07–2.62; *p* = 0.023), and diagnosis of undifferentiated tumours (HR 2.12; 95% CI, 1.45–3.08, *p* < 0.001)) as well [109]. 

Cancer-related inflammation has emerged as one of the hallmarks of cancer [110]. It has been demonstrated that CRC cells, cancer-associated fibroblasts and endothelial cells, as well as various leukocyte populations such as tumor-associated macrophages, construct a favourable microenvironment to promote tumor growth and invasiveness [111]. However, the diagnostic role of FAR (fractional albumin rate) (FAR = 100 × Fibrinogen/Albumin) and FPR (fibrinogen to prealbumin ratio) (FPR = Fibrinogen/pre-Albumin) in CRC is not completely clarified. Some reports suggested that NLR, FAR, and FPR are increased in early disease, as compared to healthy controls, and as such they could be realistically implemented for early diagnostic consideration [112]. The diagnostic utility of FAR and FPR was higher than NLR, and the combination of FPR, CEA, and CA19-9 could optimize the discrimination ability of CRC from benign disease. This is reasonable, given that serum fibrinogen, albumin and pre-albumin, which potentially are biomarkers of both inflammation and nutritional condition, were recognized predictive factors of recovery and survival of CRC patients [113,114]. In addition, the inflammatory cytokine interleukin-6 modulates the production of albumin by hepatocytes, resulting in hypoproteinaemia in CRC patients [115]. Therefore, malnutrition and immunologic impairment affect clinical outcome of patients with CRC [115].

The change in the level of acute-phase reactants, such as fibrinogen, and albumin, has been reported to be related to poor prognosis in CRC patients. However, their sensitivity and specificity alone, and combined in different cohorts, should be further investigated. Regarding this, the diagnostic efficacy of FPR and FAR for CRC should be explored through prospective research.

## 10. Stool Based Tests and Biomarkers

While fecal samples evaluate the structure of the gut flora in a non-invasive way, they do not illustrate the structure of the mucosal microbiome [116]. Mucosal biopsy samples provide the advantage of sampling healthy and diseased tissue from the same individuals. The identification of intestinal microbiota-based biomarkers has become diagnostically challenging, given their association with tumorigenesis. Regarding this, Fusobacterium has potentially been involved in tumorigenesis [117] and is often enriched in people with carcinomas; however, it is not of pathognomonic value [118].

Apart from the unmet need to identify biomarkers in patients with CRC, their identification in cases of adenomas is important in order to be potentially optimized early detection of tumors. Some studies reported that total number of taxa is lower in those with adenomas as compared to controls [119]. However, classification of normal intestine or adenomas based entirely on the bacteria present in the stool samples is not feasible. 

Importantly, the diagnosis of adenomas has been improved with the analysis of 16S rRNA gene sequence data accompanied by fecal immunochemical evaluation [120]. A meta-analysis has demonstrated that 16S rRNA gene sequences from novel candidates, such as Akkermansia, Fusobacterium, and Parvimonas, represented stool biomarkers potentially involved in carcinomas. On the other hand, adenomas had significantly higher abundances of Haemophilus, Methanosphaera, Prevotella, and Succinivibrio [121].

Further development of microbial biomarkers should be based on including both additional biomarkers, as well as larger numbers of patients. Patchy distribution of several biomarkers across individuals indicates that there are either various mechanisms causing disease or just the inflammation that can be mediated by diverse bacteria. The reproducibility and replicability of microbiota studies should be increased. However, the fact that 16S rRNA gene sequence data are not widely available is a barrier to the performance of meta-analyses.

## 11. MiRNAs

MiRNAs represent extremely stable, single stranded molecules with hairpin-loop shape and small size found in exosomes. They play a vital role in the genetic control of CRC development, progression and metastatic potential. Several studies highlight the potential utility of miRNAs as biomarkers in either tissues or blood for the assessment of response to the agents implemented in CRC, including the 5-fluorouracil based therapies, and EGFR inhibitors. Development of miRNA signature for predicting treatment response designates a personalized therapeutic approach of CRC.

The utility of serum miR-21 as a marker of CRC diagnosis and progression has been intensively investigated [122]. A large population-based study has enrolled stage II CRC patients and demonstrated that high expression of miR-21 in tumor specimens is associated with shorter DFS [123]. Apart from miR-21, the miRNAs based classifiers miR-20a-5p, miR-103a-3p, miR-106a-5p, and miR-143-5p have been reported as novel predictive markers for the recurrence of stage II CRC [124]. 

MiR-320e has been recognized as a prognostic biomarker in CRC based on the evaluation of two separate cohorts of patients treated with FOLFOX [125]. Regarding this, it was revealed that the high level of miR-320e in primary CRC samples is correlated with more advanced disease, recurrence, and dismal prognosis in the subgroup of patients with stage II and III CRC. Furthermore, there is some evidence for contribution of miR-148a to the carcinogenesis of CRC. Downregulation of miR-148a expression in primary tissues is associated with the development of high grade adenoma and promotes disease progression [126]. Consequently, miR-148a potentially represents a predictive biomarker for the FOLFOX regime. Similarly, postoperative plasma miR-31, miR-141, and miR-16 are suggested biomarkers of disease recurrence after the surgical resection [127]. It has also been reported that miR-429 expression is upregulated in CRC tissue and as such closely related to tumor size, lymph node involvement, and distant metastases, whereas it leads to shorter survival [128]. 

At the same time, miRNAs have been found to induce chemoresistance. Indeed, FOLFOX-resistance in advanced CRC is significantly associated with upregulation and downregulation of several serum miRNAs (Table 2) [125,128,129,130,131,132,133,134,135,136,137,138,139,140,141,142,143]. The differentiation of FOLFOX-resistant from FOLFOX responsive patients by serum miR-19a had a reported sensitivity and specificity of 66.7 and 63.9%, respectively [137]. In terms of treatment response to anti-VEGF or anti-EGFR inhibitors in metastatic CRC, upregulation of miR-126 was correlated with bevacizumab resistance [144], whereas overexpression of miR-31, miR-100, miR-125b, and downregulation of miR-7, with resistance to cetuximab, respectively [145,146,147]. Table 2 summarizes the miRNAs involved in resistance to chemotherapeutic as well as molecular targeted agents.

There is a trend toward development of a next-generation diagnostic panel of miRNAs. Furthermore, discovery and characterization of new mRNA will explore therapeutic targets to overcome drug resistance. Nevertheless, several obstacles limit their application in clinical practice. The different selection criteria for patients, collection methods and processing of biological samples may contribute to the different miRNA signatures obtained. As such, the clinical use of miRNA as biomarkers is still limited due to the inconsistency and limited reproducibility among the published studies so far. Therefore, an optimal approach for miRNA detection should be followed, focusing on the variability in patients’ characteristics, experimental design, as well as the isolation and detection methodologies. Design of large-scale prospective studies is essential for the validation of these potential prognostic and predictive biomarkers, and determination of their levels. The specificity and sensitivity of each individual miRNA as a biomarker could be optimized, when their evaluation is accompanied by the conventional biomarkers, such as CEA. 

## 12. Differences in Genomics between Right and Left Primary Tumors

From an anatomical and surgical point of view, there is no uniform distinction between right- and left-sided CRC. Nevertheless, the right-sided colon comprises the ileocecum, the ascending colon, and the transverse colon, whereas the left-sided colon contains the descending and the sigmoid colon [168]. The different ontogenesis within CRC proposes biological discrimination between right- and left-sided CRC. There are alterations in patterns of DNA methylation between the right and left colon [169], and evidence of epigenetic aberrations in preneoplastic right colon mucosa [170]. Despite its rarity in metastatic settings, MSI-H CRC is more frequent in the right colon. Therefore, MSI assessment should become a standard practice in metastatic CRC, given that an immune checkpoint blockade is related to high RR in MSI-H cases [28].

Prospective clinical trials suggested that right-sided CRC is associated with a significantly worse prognosis compared to left-sided tumors [171,172]. In addition, right-sided cancers predict a lack of response to EGFR inhibition [173]. Among microsatellite stable metastatic CRC, a right-sided disease is most likely to harbor either a hotspot *RAS* mutation or *BRAF V600E*, as compared to left-sided (80% vs. 46%, respectively). Overall, the dismal prognosis of the right-sided tumors is related to their higher rate of mitogenic mutations, and more common *RAS*, *PI3K*, and *TGFβ* pathway alterations. As far as left-sided CRC is concerned, mutations in *APC* and *TP53*, as well as amplifications of receptor tyrosine kinases, including *HER2* and *EGFR*, are more frequent as compared to right-sided tumors [174]. Similarly, there is high left-sided tumor expression of *EGFR* ligands AREG and EREG, which lead to high sensitivity to EGFR blockades [175]. In contrast, tumor sidedness does not seem to have a predictive role in terms of therapeutic approaches based on the anti-angiogenic agent bevacizumab [176]. 

## 13. Conclusions

Colorectal cancer is a highly heterogeneous clinical entity, and pathological evaluation is a suboptimal method for the consideration of disease prognosis. Multiple time point sequencing for the evaluation of the clonal development process, treatment response, and resistance throughout the course of CRC are hugely important. In this era, recent advances in the identification of molecular signatures contribute to the development of novel therapeutic strategies. MSI-H tumors are potentially responsive to immunotherapy, particularly the PD-1 blockades, as they are found to be infiltrated by lymphocytes. The utilization of the *KRAS*, *NRAS*, and *BRAF* genes as prognostic and predictive biomarkers is an important step toward a personalized therapeutic approach of CRC patients. Aberrant miRNA expression may serve as a biomarker as well, despite the fact that an optimal strategy for their detection and validation is still required. 

Adaptive clinical trials should be designed for the determination of real-time pharmacodynamic markers. They could be integrated into a prediction model for the evaluation of the benefit from either adjuvant chemotherapy, or targeted therapies for metastatic CRC.

## Figures and Tables

**Table 1:** Summary of studies investigated the effect of targetable genomic alterations on survival in metastatic CRC.

Alteration	Therapy	Number of Patients	ORR (%)	Median DFS (Months)	Author/Reference	Year of Publication
BRAF V600E	D + T + P	83	18	NR	Corcoran et al./[34]	2018
	V + I + C	19	35	7.7	Hong et al./[35]	2016
	V	21	5	2.1	Kopetz et al./[36]	2015
	V + P	15	13	3.2	Yaeger et al./[37]	2015
	D + T	43	12	3.5	Corcoran et al./[38]	2015
	V	10	0	4.5	Hyman et al./[39]	2015
	V + C	27	23	3.7		
HER2 amplification	Tras + Pert	34	38	NR	Hainsworth et al./[40]	2018
	Tras + Lap	27	30	5	Sartore-Bianchi et al./[41]	2016
NTRK fusion	Laro	4	50	NA	Drilon et al. [42]	2018
ALK fusion	Ceritinib	1	NA	NA	Yakirevich et al./[43]	2016

CRC, colorectal cancer; ORR, objective response rate; DFS, disease-free survival; D, dabrafenib; T, trametinib; P, panitumumab; NR, not reached; V, vemurafenib; I, irinotecan; C, cetuximab; Tras, trastuzumab; Pert, pertuzumab; Lap, lapatinib; Laro, larotrectinib; NA, not applicable.

**Table 2:** miRNAs functions in therapeutic resistance in CRC.

miRNA	Drug(s) Affected	Effect on Drug Resistance	Number of Patients	Author/Reference	Year of Publication
miR-139-5p	FOLFOX	Upregulation	250	Miyoshi et al./[129]	2017
miR-92b-3p, miR-3156-5p, miR-10a-5p, and miR-125a-5p			61	Kiss et al./[130]	2017
miR-429			78	Dong et al./[128]	2016
miR-425-5p			-	Zhang et al./[131]	2016
miR-320e			100	Perez-Carbonell et al./[125]	2015
miR-520g			-	Zhang et al./[132]	2015
miR-17-5p			100	Fang et al./[133]	2014
miR-106a, miR-130b, and miR-484			150	Kjersem et al./[134]	2014
miR-20a, miR-130, miR-145, miR-216, and miR-372			40	Zhang et al./[135]	2014
miR-155			15	Chen et al./[136]	2014
miR-19a			72	Chen et al./[137]	2013
miR-27b, miR-181b, and miR-625-3p			257	Rasmussen et al./[138]	2013
miR-148a			273	Takahashi et al./[139]	2012
miR-20a			-	Chai et al./[140]	2011
miR-34a		Downregulation	30	Sun et al./[141]	2017
miR-218			116	Li et al./[142]	2017
miR-4772-3p			84	Liu et al./[118,143]	2016
miR-195	5FU	Upregulation	-	Kim et al./[148]	2018
miR-224			12	Amankwatia et al. / [149]	2015
miR-587			19	Zhang et al./[135,150]	2015
miR-23a			38	Shang et al./[151]	2014
miR-10b			88	Nishida et al./[152]	2012
miR-19b			-	Kurokawa et al./[153]	2012
miR-21			76	Valeri et al./[154]	2010
miR-31			-	Wang et al./[155]	2010
miR-215			24	Song et al./[156]	2010
miR-140			24	Song et al./[157]	2009
miR-761		Downregulation	28	Cao et al./[158]	2017
miR-18a*/miR-4802			123	Yu et al./[159]	2017
miR-203			-	Li et al./[160]	2015
miR-203	Oxaliplatin	Upregulation	-	Zhou et al./[161]	2014
miR-200b-3p		Downregulation	97	Lv et al./[162]	2017
miR-141/miR-200c			1	Tanaka et al./[163]	2015
miR-153			100	Zhang et al./[164]	2013
miR-1915			-	Xu et al./[165]	2013
miR-194	Oxaliplatin, irinotecan	Downregulation	70	Chang et al./[166]	2017
Let-7g	S-1 (Tegafur/gimeracil/oteracil)	Upregulation	46	Nakajima et al./[167]	2006
miR-181b	S-1, 5-FU				
miR-100/miR-125b	Cetuximab	Upregulation	10	Lu et al./[146]	2017
miR-31			93	Mlcochova et al./[145]	2015
miR-7		Downregulation	105	Suto et al./[147]	2015
miR-126	Bevacizumab	Upregulation	68	Hansen et al./[144]	2015

CRC, colorectal cancer; FOLFOX, 5-fluorouracil, leucovorin, and oxaliplatin; 5FU, 5-fluorouracil.
